# Supplementary material for: Neuronal wiring diagram of an adult brain
Source: Nature. 2024 Oct 2;634(8032):124–38. doi: 10.1038/s41586-024-07558-y (PMC11446842; doi:10.1038/s41586-024-07558-y)
Supplement: Supplementary file 1 — This file contains Supplementary Figs. 1–10, Supplementary Tables 1–3 and legends for Supplementary Video files. [file 41586_2024_7558_MOESM1_ESM.docx]

**
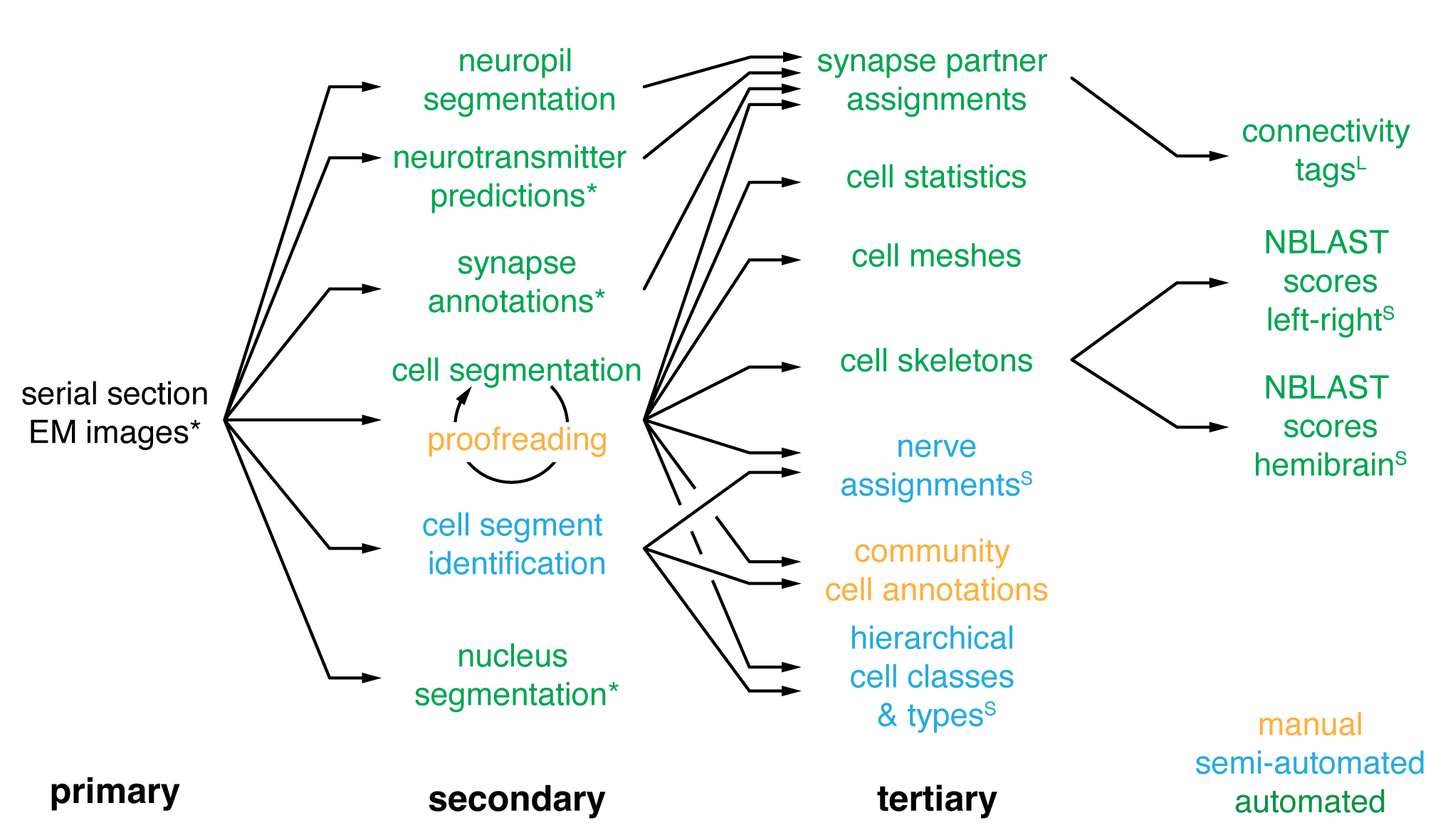
Supplementary Figure 1 | Flow of data products.** All data was derived from the original EM images but some data resources built on intermediate products. This diagram shows how different data products build on each other. Data products annotated with a “*” were published previously and made publicly available^7,9,10^. Those annotated with an “S” are described in more detail by Schlegel et al.^12^**,** those annotated with an “L” are described in Lin et al**.^40^.**

**
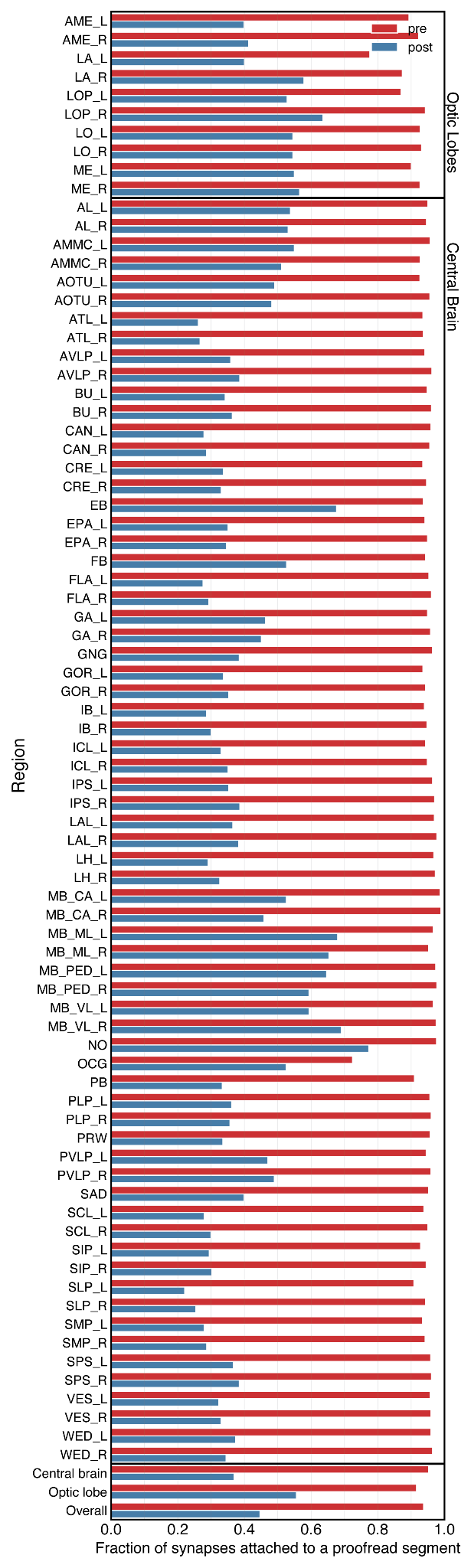
**

**Supplementary Figure 2** | **Completion rates by neuropil.**

**
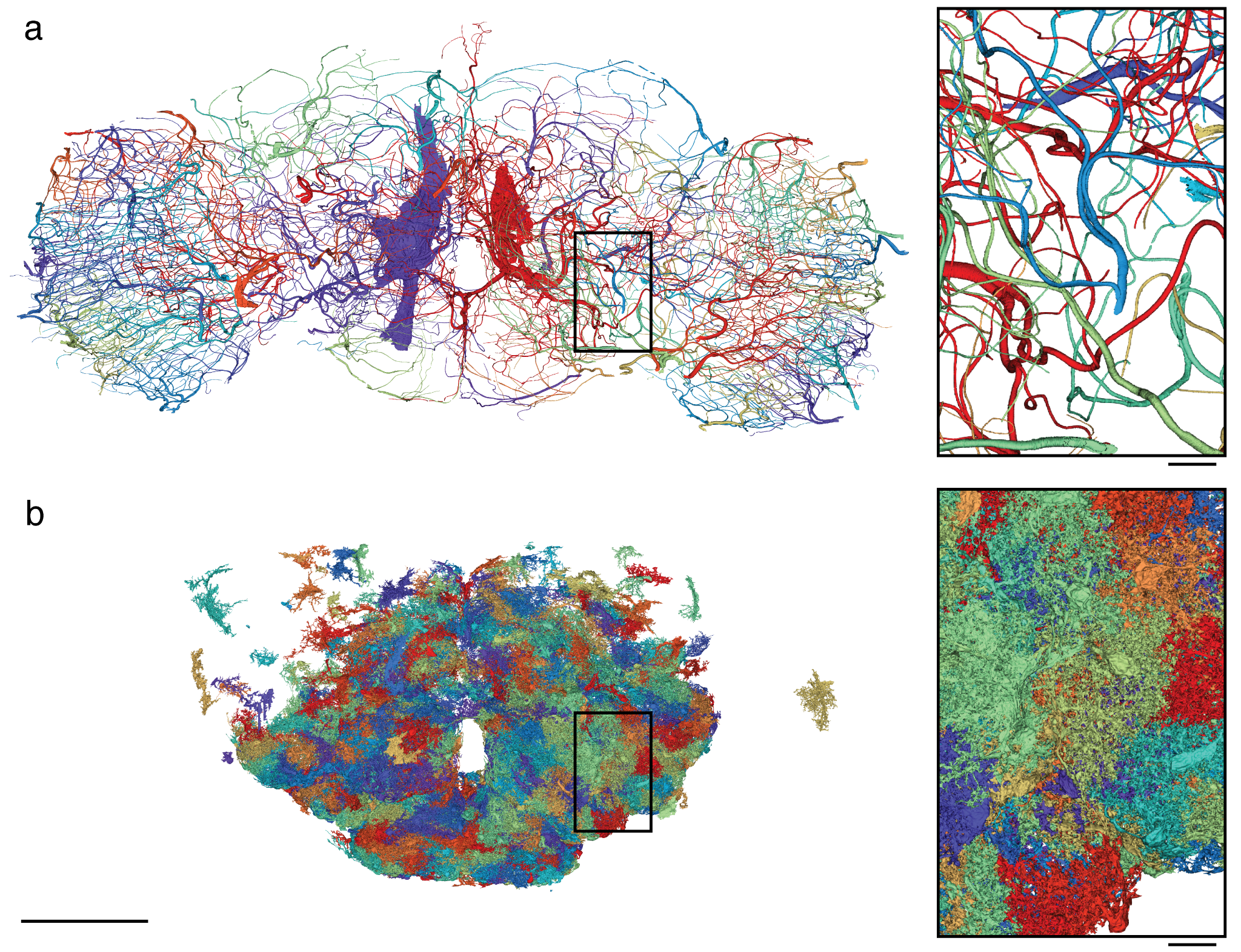
**

**Supplementary Figure 3 | Trachea and glia cells.** (a) Rendering of all trachea segments in the FlyWire dataset. (b) Rendering of some reconstructed glia cells in the FlyWire dataset. At the time of writing, only a subset of the glia cells, with bias towards the central brain, have been proofread and labeled. Scale bar: 100 µm; insets: 10 µm.
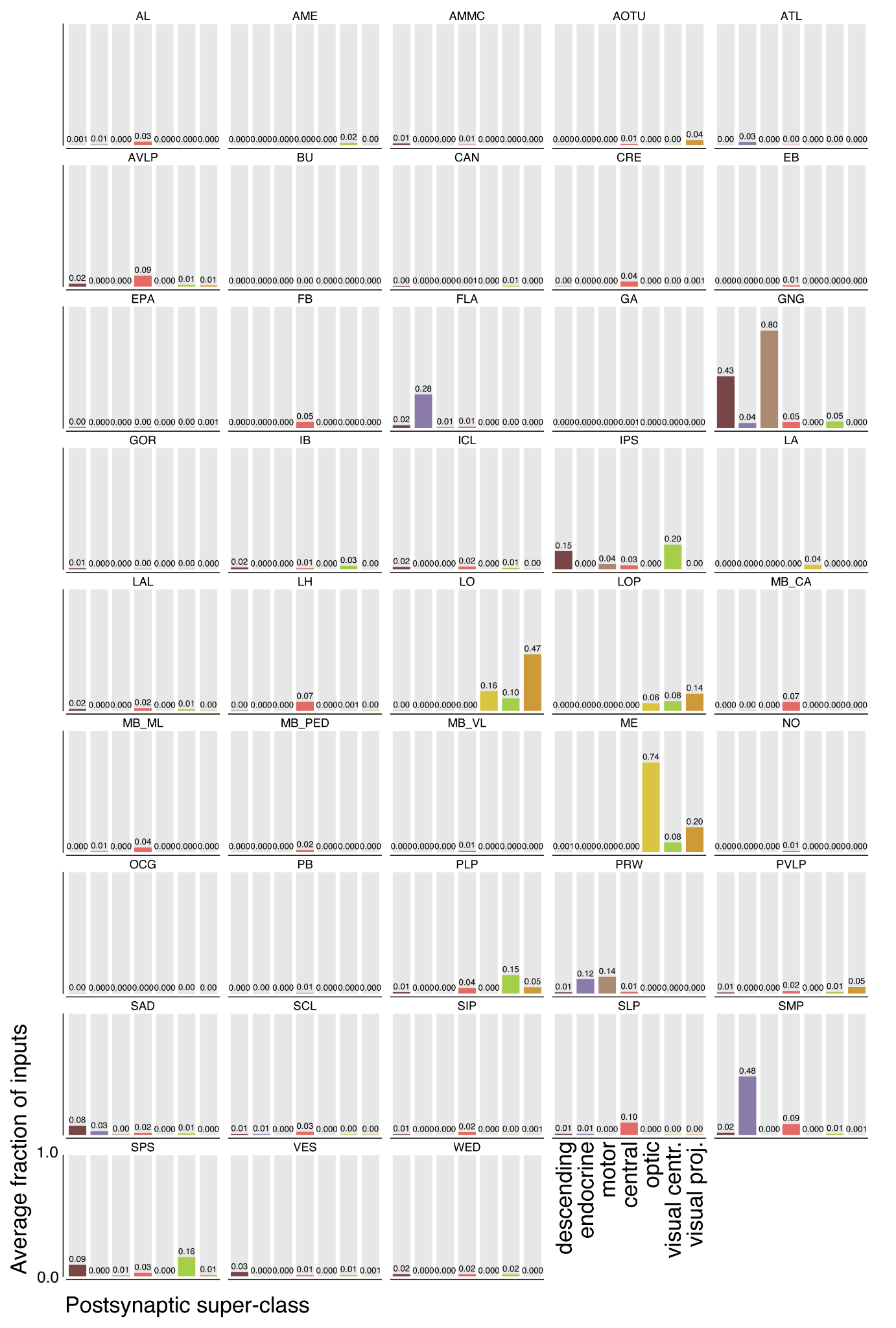


**Supplementary Figure 4 | Average input fraction for each super-class by neuropil.** Average input fractions were computed by summing the row values for each neuropil in the super-class specific projection matrices. Mirrored neuropils were added to one.


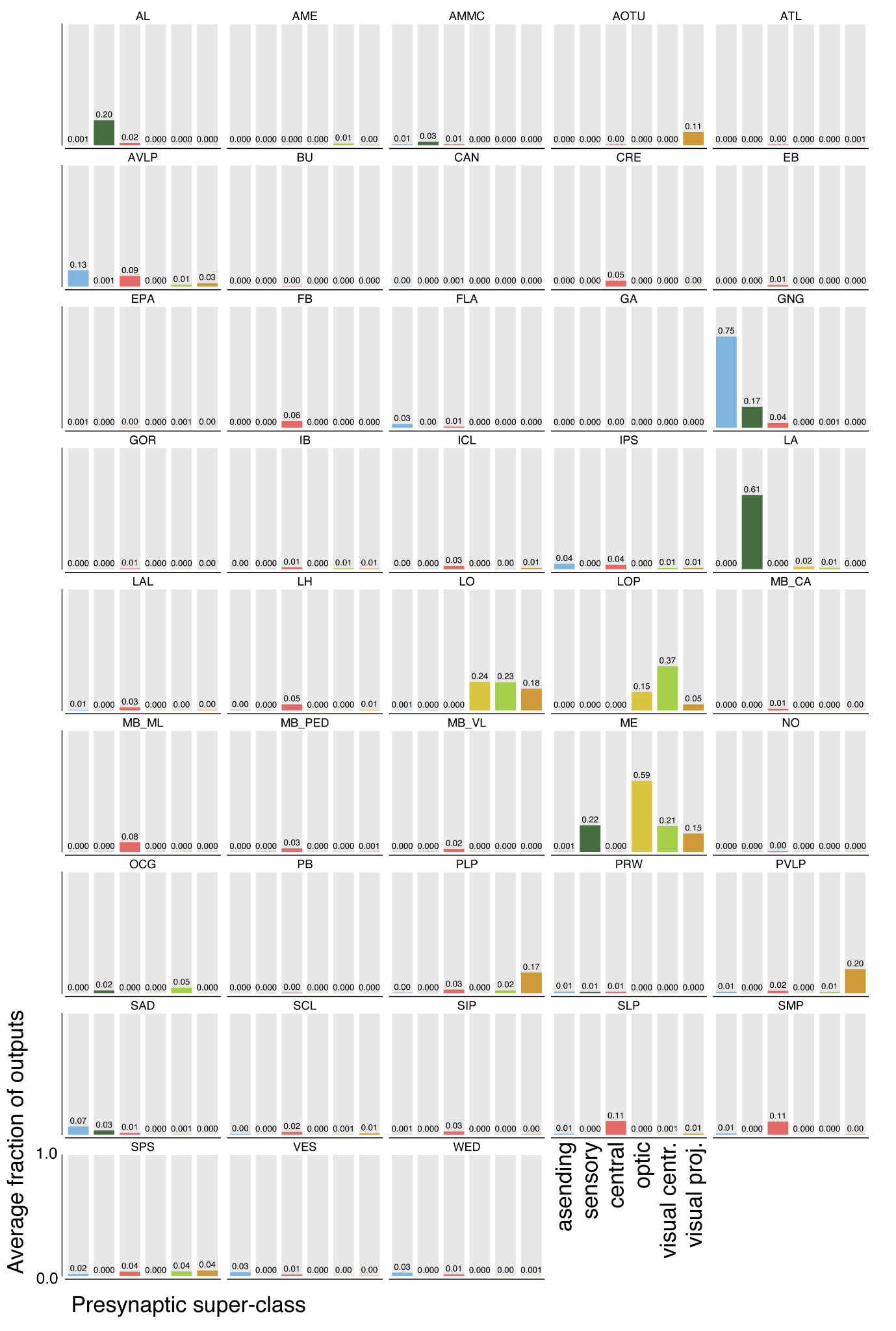


**Supplementary Figure 5 | Average output fraction for each super-class by neuropil.** Average output fractions were computed by summing the column values for each neuropil in the super-class specific projection matrices. Mirrored neuropils were added to one.


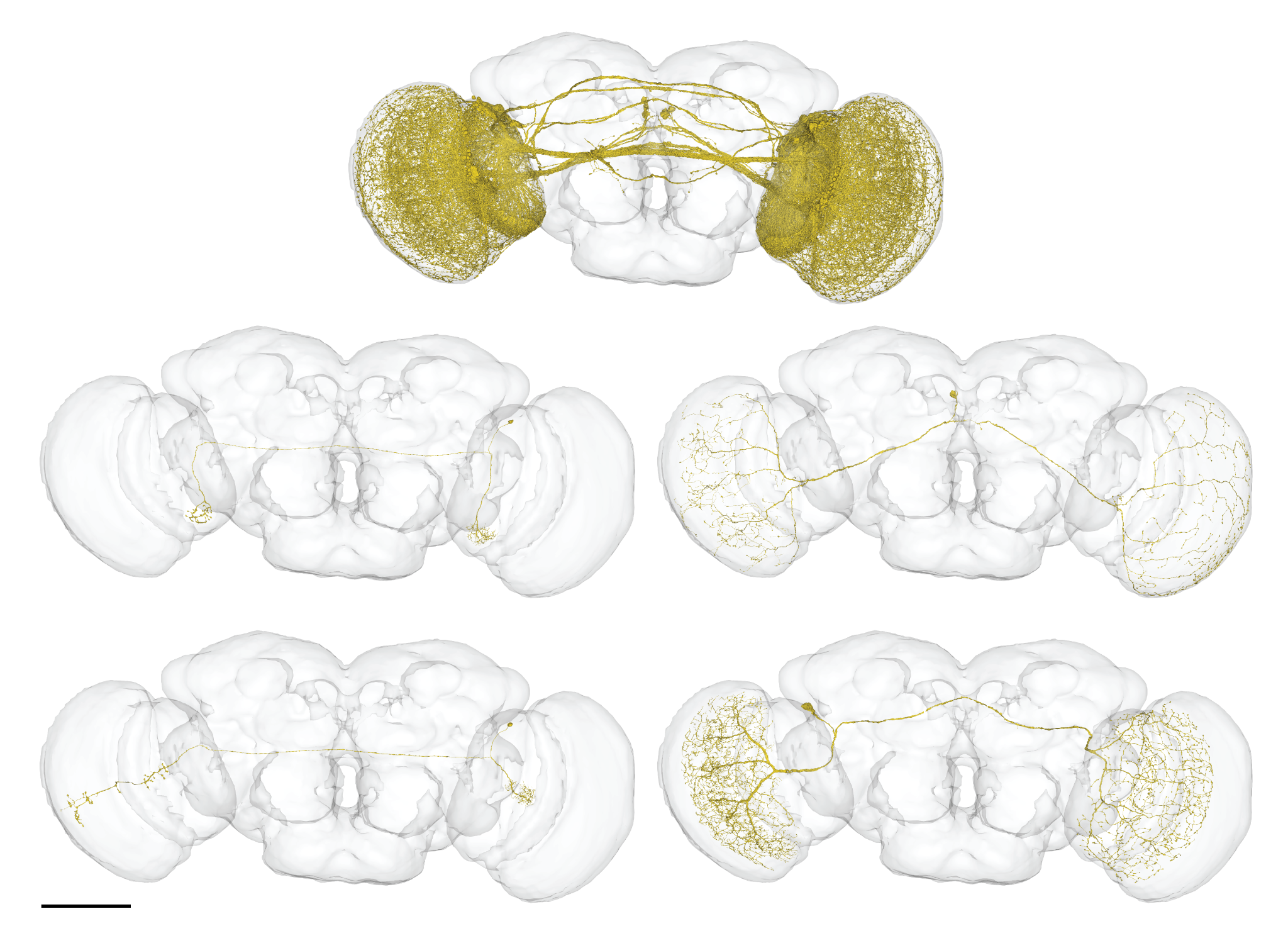


**Supplementary Figure 6 | Bilateral optic lobe neurons.** On the left: putative LC14 (top) and putative LC14b (bottom). Scale bar: 100 µm


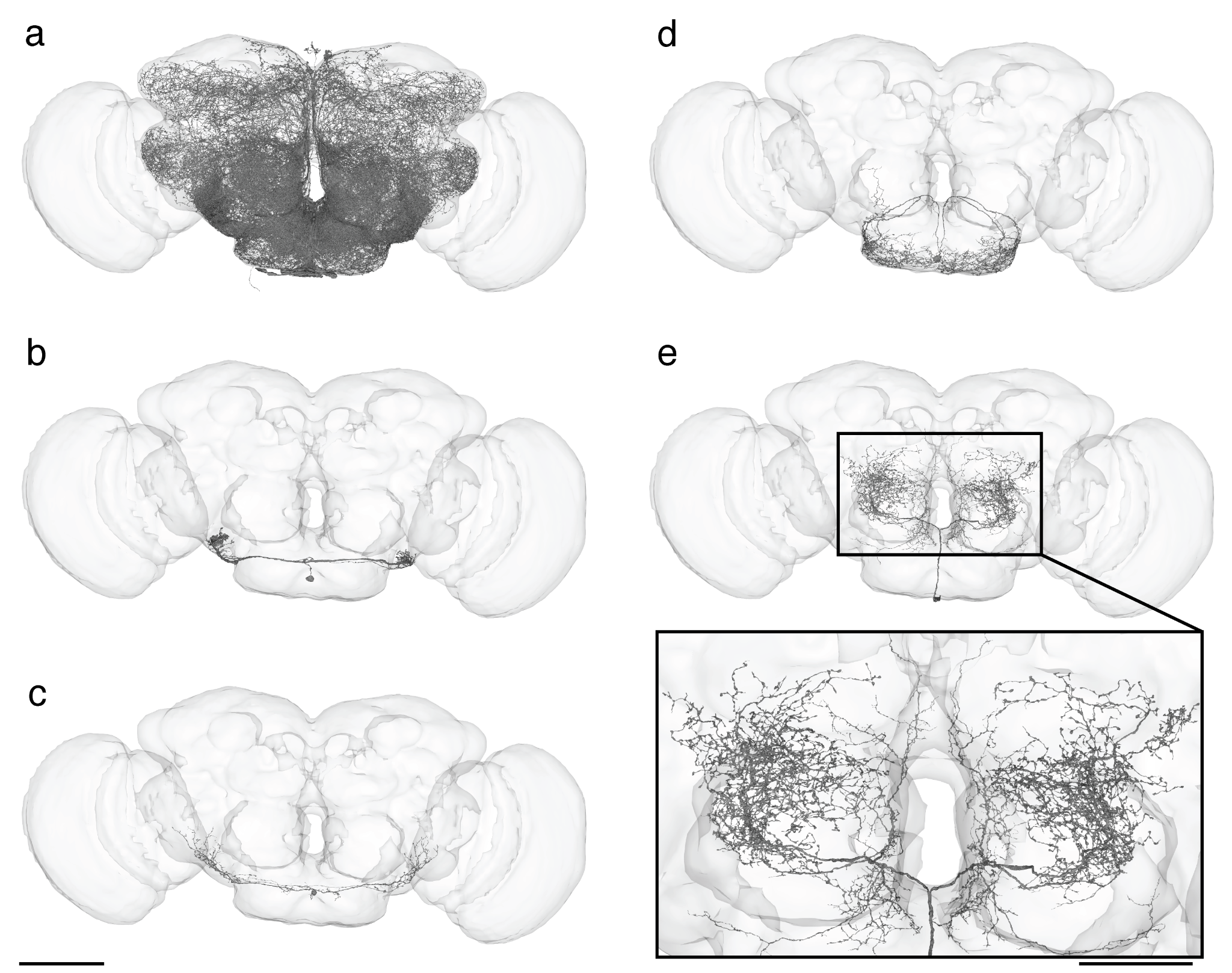


**Supplementary Figure 7 | Neurons on the midline with dendrites in both hemispheres.** (a) All symmetric neurons with a cell body on the midline. (b-e) examples of individual neurons. Scale bar: 100 µm, inset: 50 µm


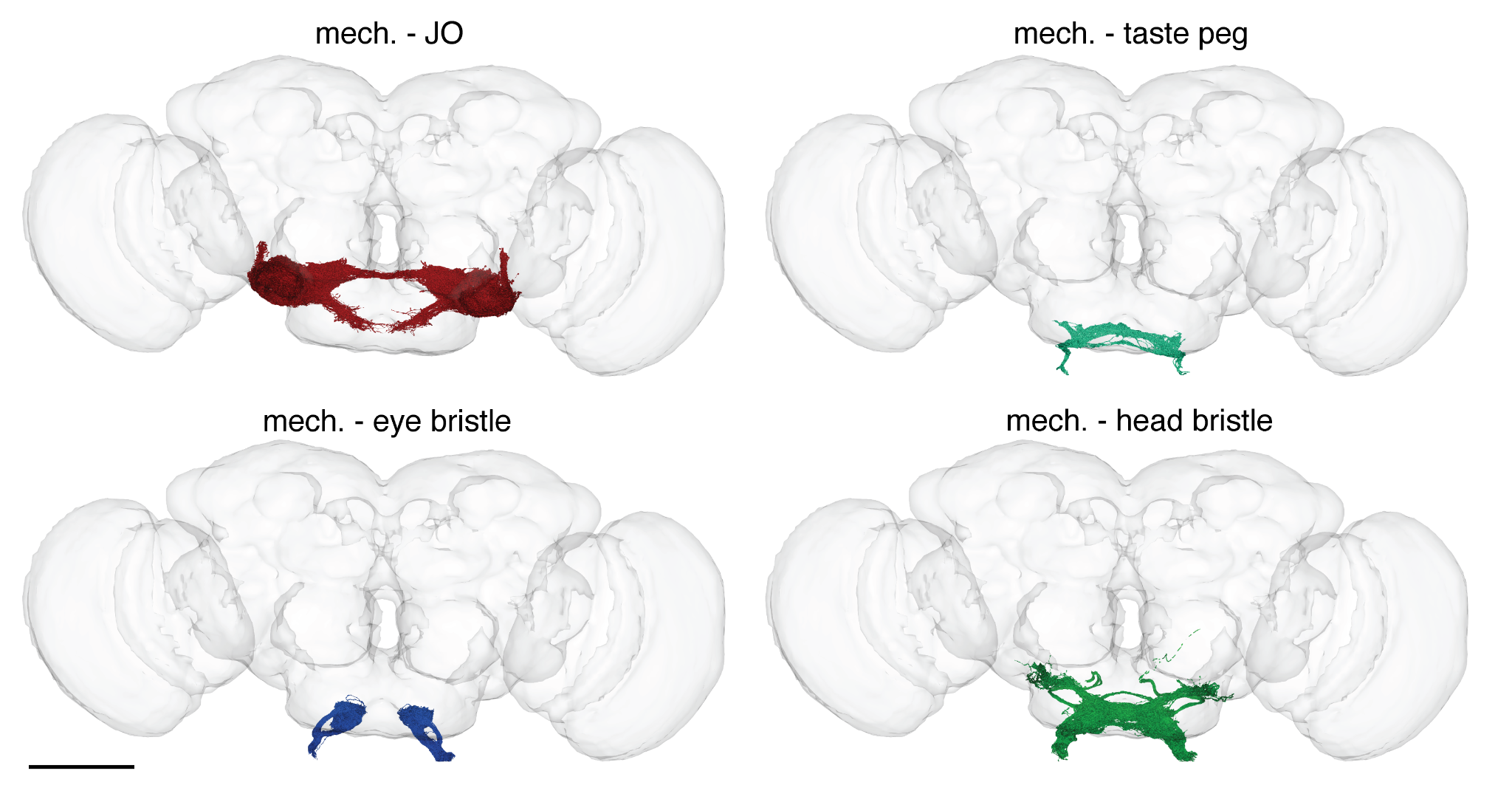


**Supplementary Figure 8 | Mechanosensory neuron subtypes.** Scale bar: 100 µm

**
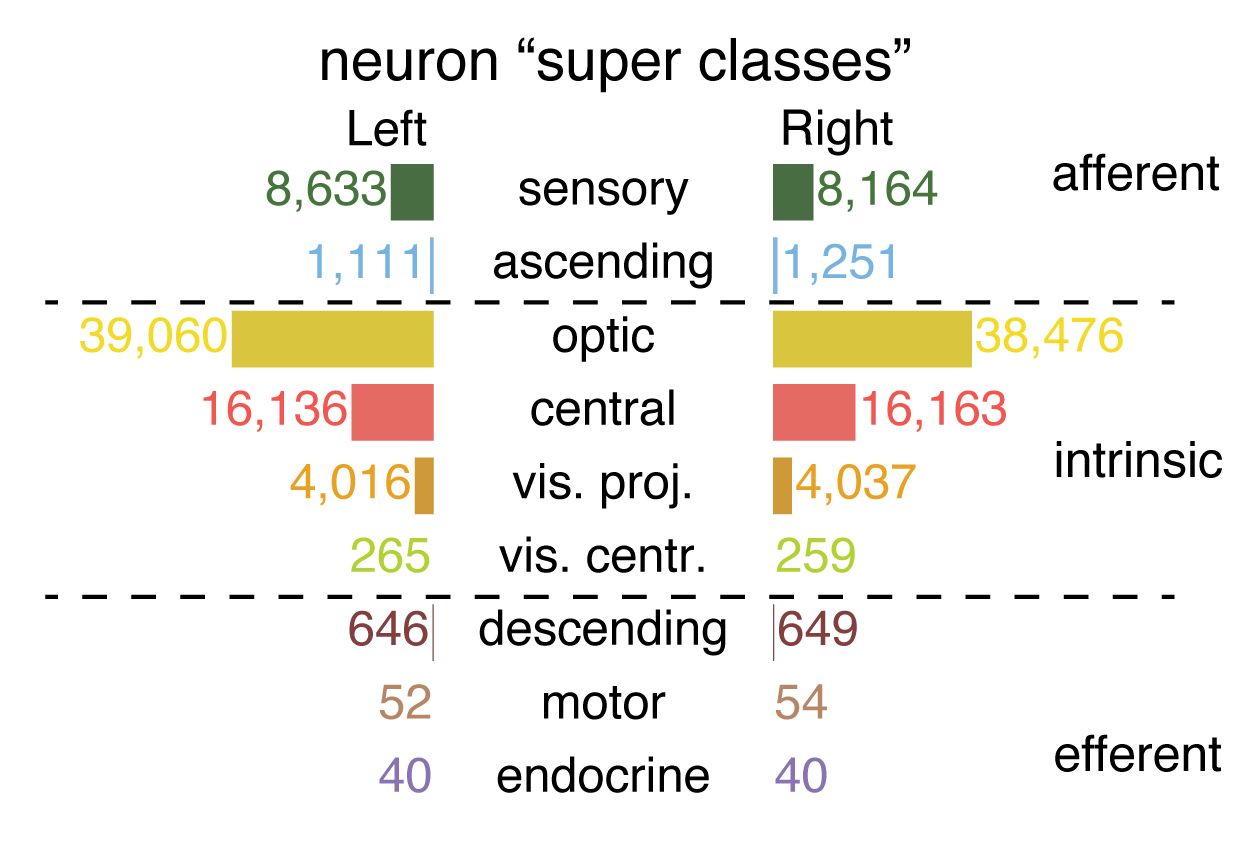
**

**Supplementary Figure 9 | Distribution of community annotations by super class**


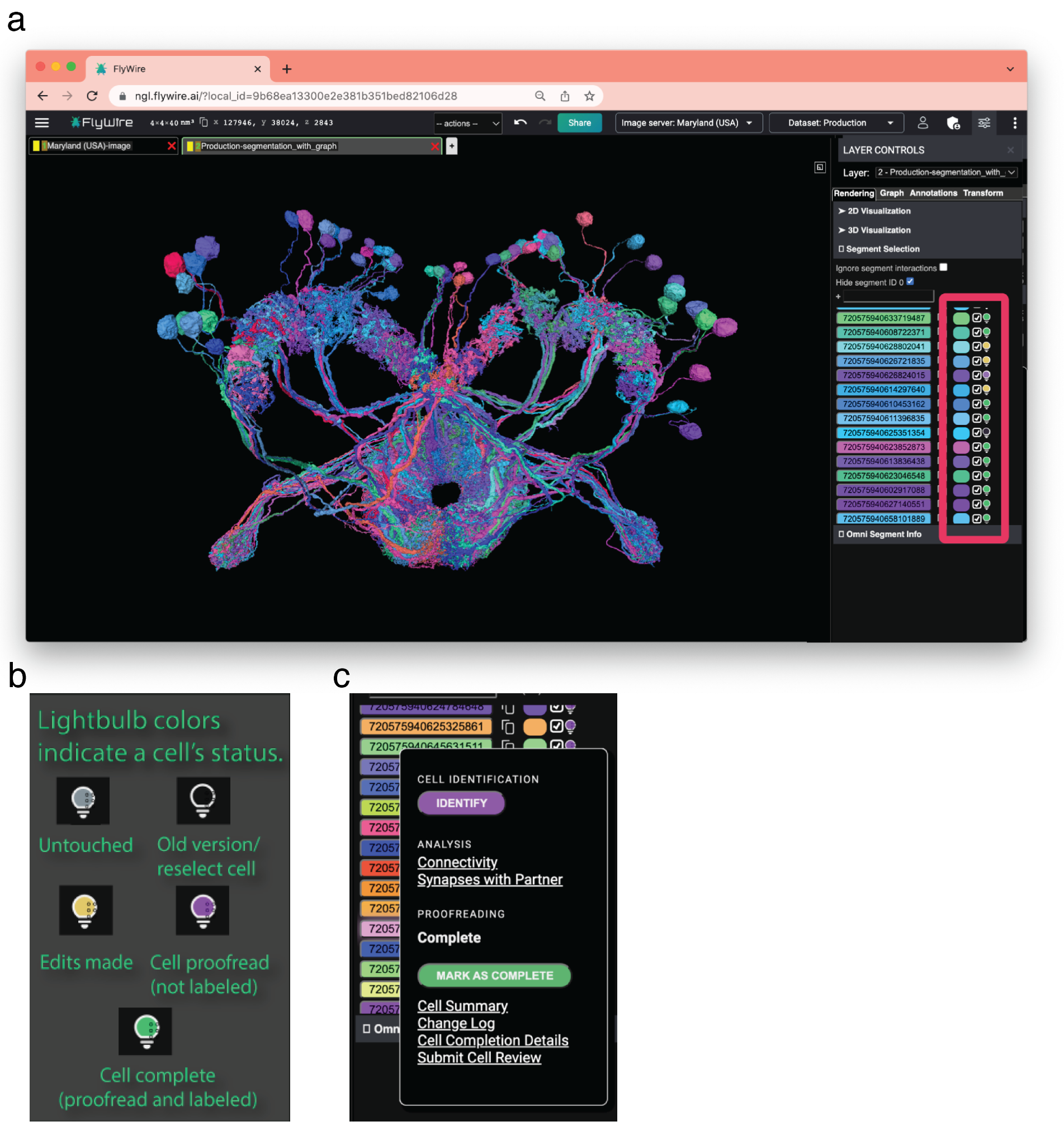


**Supplementary Figure 10 | Neuroglancer Interface and Lightbulb.** (a) FlyWire's lightbulb menu displays the proofreading and annotation status of every segment (red box). (b) It is color coded for easy reference (yellow: cell has not been declared complete; purple: complete but not labeled; green: proofread and labeled; black: out of date segmentation). (c) Users can load cell identification directly within the FlyWire editor, perform basic connectivity analysis, and view a cell's edit history.

| **Name** | **Lab Affiliation** | **total edits** | **specific contributions** |
| --- | --- | --- | --- |
| Doug Bland | Mala Murthy Lab, Sebastian Seung Lab | 295,202 |  |
| Zairene Lenizo | Mala Murthy Lab, Sebastian Seung Lab | 127,401 |  |
| Nseraf | Flyers (citizen scientist) | 110,082 |  |
| Nashra Hadjerol | Mala Murthy Lab, Sebastian Seung Lab | 92,883 |  |
| Kyle Patrick Willie | Mala Murthy Lab, Sebastian Seung Lab | 92,695 |  |
| Austin T Burke | Mala Murthy Lab, Sebastian Seung Lab | 85,953 |  |
| Ryan Willie | Mala Murthy Lab, Sebastian Seung Lab | 80,595 |  |
| John Anthony Ocho | Mala Murthy Lab, Sebastian Seung Lab | 75,794 |  |
| Joshua Bañez | Mala Murthy Lab, Sebastian Seung Lab | 69,225 |  |
| Rey Adrian Candilada | Mala Murthy Lab, Sebastian Seung Lab | 60,158 |  |
| Nelsie Panes | Mala Murthy Lab, Sebastian Seung Lab | 59,560 |  |
| Arti Yadav | Greg Jefferis Lab | 59,394 |  |
| Shirleyjoy Serona | Mala Murthy Lab, Sebastian Seung Lab | 57,085 |  |
| Yijie Yin | Greg Jefferis Lab | 50,858 |  |
| Jet Ivan Dolorosa | Mala Murthy Lab, Sebastian Seung Lab | 50,655 |  |
| Remer Tancontian | Mala Murthy Lab, Sebastian Seung Lab | 46,931 |  |
| Ariel Dagohoy | Mala Murthy Lab, Sebastian Seung Lab | 46,169 |  |
| Regine Salem | Mala Murthy Lab, Sebastian Seung Lab | 45,090 |  |
| Kendrick Joules Vinson | Mala Murthy Lab, Sebastian Seung Lab | 44,771 |  |
| Mendell Lopez | Mala Murthy Lab, Sebastian Seung Lab | 43,885 |  |
| Laia Serratosa Capdevila | Greg Jefferis Lab, Rachel Wilson Lab | 41,723 |  |
| Griffin Badalamente | Greg Jefferis Lab | 41,674 |  |
| Krzysztof Kruk | Flyers (citizen scientist) | 40,752 |  |
| Anjali Pandey | Greg Jefferis Lab | 37,344 |  |
| Ben Silverman | Mala Murthy Lab, Sebastian Seung Lab | 37,016 |  |
| Darrel Jay Akiatan | Mala Murthy Lab, Sebastian Seung Lab | 36,987 |  |
| Dustin Garner | Sung Soo Kim Lab | 36,815 |  |
| Dharini Sapkal | Greg Jefferis Lab | 31,244 |  |
| annkri (Anne Kristiansen) | Flyers (citizen scientist) | 29,345 |  |
| Jay Gager | Mala Murthy Lab, Sebastian Seung Lab | 29,242 |  |
| Shaina Mae Monungolh | Mala Murthy Lab, Sebastian Seung Lab | 28,721 |  |
| Miguel Albero | Mala Murthy Lab, Sebastian Seung Lab | 26,831 |  |
| Mark Lloyd Pielago | Mala Murthy Lab, Sebastian Seung Lab | 26,831 |  |
| Jacquilyn Laude | Mala Murthy Lab, Sebastian Seung Lab | 26,006 |  |
| Katharina Eichler | Greg Jefferis Lab, Seeds Hampel Lab | 24,481 |  |
| Zeba Vohra | Greg Jefferis Lab | 24,431 |  |
| Allien Mae Gogo | Mala Murthy Lab, Sebastian Seung Lab | 24,202 |  |
| Emil Kind | Mathias Wernet Lab | 24,062 |  |
| Alvin Josh Mandahay | Mala Murthy Lab, Sebastian Seung Lab | 24,062 |  |
| Varun Sane | Greg Jefferis Lab | 23,762 |  |
| Chereb Martinez | Mala Murthy Lab, Sebastian Seung Lab | 22,540 |  |
| John David Asis | Mala Murthy Lab, Sebastian Seung Lab | 21,894 |  |
| Chitra Nair | Greg Jefferis Lab | 21,696 |  |
| Márcia dos Santos | Greg Jefferis Lab | 21,610 |  |
| Dhwani Patel | Greg Jefferis Lab | 20,389 |  |
| Marchan Manaytay | Mala Murthy Lab, Sebastian Seung Lab | 20,277 |  |
| Thomas Stocks | Flyers (citizen scientist) | 20,176 |  |
| Imaan F. M. Tamimi | Greg Jefferis Lab | 19,937 |  |
| Clyde Angelo Lim | Mala Murthy Lab, Sebastian Seung Lab | 19,877 |  |
| James Hebditch | Mala Murthy Lab, Sebastian Seung Lab | 19,666 |  |
| Philip Lenard Ampo | Mala Murthy Lab, Sebastian Seung Lab | 19,132 |  |
| Michelle Darapan Pantujan | Mala Murthy Lab, Sebastian Seung Lab | 18,915 |  |
| Alexandre Javier | Greg Jefferis Lab | 17,813 |  |
| Daril Bautista | Mala Murthy Lab, Sebastian Seung Lab | 17,555 |  |
| Rashmita Rana | Greg Jefferis Lab | 17,547 |  |
| Jansen Seguido | Mala Murthy Lab, Sebastian Seung Lab | 17,407 |  |
| Bhargavi Parmar | Greg Jefferis Lab | 17,133 |  |
| John Clyde Saguimpa | Mala Murthy Lab, Sebastian Seung Lab | 16,656 |  |
| Merlin Moore | Mala Murthy Lab, Sebastian Seung Lab | 16,590 |  |
| AzureJay (Jaime Skelton) | Flyers (citizen scientist) | 15,825 |  |
| Mark Larson | Wei-Chung Lee Lab | 15,692 |  |
| Joseph Hsu | Greg Jefferis Lab, Scott Waddell Lab | 15,496 |  |
| Itisha Joshi | Greg Jefferis Lab | 14,717 |  |
| Dhara Kakadiya | Greg Jefferis Lab | 14,544 |  |
| Cathy Pilapil | Mala Murthy Lab, Sebastian Seung Lab | 13,558 |  |
| Kaushik Parmar | Greg Jefferis Lab | 13,136 |  |
| Philipp Schlegel | Greg Jefferis Lab | 12,846 |  |
| Irene Salgarella | Greg Jefferis Lab | 12,475 |  |
| Chan Hyuk Kang | Jinseop Kim Lab | 11,129 |  |
| Markus William Pleijzier | Greg Jefferis Lab | 10,498 | Reconstruction of Mushroom Body neurons, Lateral Horn Neurons and Lateral Horn Centrifugal Neurons |
| Marina Gkantia | Greg Jefferis Lab | 10,260 |  |
| Jinmook Lee | Jinseop Kim Lab | 9,887 |  |
| Quinn Vanderbeck | Rachel Wilson Lab | 8,845 |  |
| Yashvi Patel | Greg Jefferis Lab | 8,485 |  |
| Eva Munnelly | Greg Jefferis Lab | 8,120 |  |
| Olivia Sato | Wei-Chung Lee Lab | 8,055 |  |
| Siqi Fang | Greg Jefferis Lab | 7,981 |  |
| Janice Salocot | Mala Murthy Lab, Sebastian Seung Lab | 7,463 |  |
| Paul Brooks | Greg Jefferis Lab | 6,840 |  |
| Claire E. McKellar | Mala Murthy Lab, Sebastian Seung Lab | 6,802 |  |
| Christopher Dunne | Greg Jefferis Lab | 6,308 |  |
| Mai Bui | Ken Colodner Lab | 6,228 |  |
| JousterL (Matthew Lichtenberger) | Flyers (citizen scientist) | 5,883 |  |
| edmark tamboboy | Mala Murthy Lab, Sebastian Seung Lab | 5,801 |  |
| Mareike Selcho | Mareike Selcho Lab | 5,756 |  |
| Lucia Kmecova | Seeds Hampel Lab | 5,665 |  |
| Katie Molloy | Wei-Chung Lee Lab | 5,492 |  |
| Alexis E Santana-Cruz | Seeds Hampel Lab | 5,274 |  |
| Farzaan Salman | Andrew Dacks Lab | 5,092 |  |
| Steven Calle | Seeds Hampel Lab | 4,927 |  |
| Kfay | Flyers (citizen scientist) | 4,886 |  |
| Seongbong Yu | Jinseop Kim Lab | 4,832 |  |
| Arzoo Diwan | Greg Jefferis Lab | 4,787 |  |
| Celia David | Mala Murthy Lab, Sebastian Seung Lab | 4,546 |  |
| Monika Patel | Greg Jefferis Lab | 4,482 |  |
| Gregory S.X.E. Jefferis | Greg Jefferis Lab | 4,474 |  |
| Szi-chieh Yu | Mala Murthy Lab, Sebastian Seung Lab | 4,468 |  |
| Sarah Morejohn | Mala Murthy Lab, Sebastian Seung Lab | 4,090 |  |
| Sanna Koskela | Michael Reiser Lab | 3,822 |  |
| bl4ckscor3 (Daniel Lehmann) | Flyers (citizen scientist) | 3,735 |  |
| Sangeeta Sisodiya | Greg Jefferis Lab | 3,493 |  |
| Tansy Yang | Janelia | 3,422 |  |
| Selden Koolman | Mala Murthy Lab, Sebastian Seung Lab | 3,384 |  |
| Christa Baker | Mala Murthy Lab | 3,381 |  |
| Gerit A. Linneweber | Gerit Linneweber Lab | 3,237 |  |
| Amalia Braun | Alexander Borst Lab | 3,125 |  |
| Marissa Sorek | Mala Murthy Lab, Sebastian Seung Lab | 3,030 |  |
| Sky Cho | Ken Colodner Lab | 2,972 |  |
| Wolf Huetteroth | Wolf Huetteroth Lab | 2,846 |  |
| Brian Reicher | Wei-Chung Lee Lab | 2,794 |  |
| TR77 | Flyers (citizen scientist) | 2,775 |  |
| Marlon Blanquart | Greg Jefferis Lab | 2,662 |  |
| Hyungjun Choi | Jae Young Kwon Lab | 2,373 |  |
| Li Guo | Julie Simpson Lab | 2,095 |  |
| Forrest Collman | Forrest Collman Lab | 2,016 |  |
| Joanna Eckhardt | Mala Murthy Lab | 1,995 |  |
| Alisa Poh | Barry Dickson Lab | 1,922 |  |
| Marina Lin | Ken Colodner Lab | 1,920 |  |
| Stefanie Hampel | Seeds Hampel Lab | 1,645 |  |
| Wes Murfin | Citizen scientist | 1,578 |  |
| Peter Gibb | Rachel Wilson Lab | 1,448 |  |
| Zhihao Zheng | Sebastian Seung Lab | 1,421 |  |
| Nidhi Patel | Greg Jefferis Lab | 1,394 |  |
| Lucy Houghton | Sung Soo Kim Lab | 1,357 |  |
| Álvaro Sanz Díez | Rudy Behnia Lab | 1,317 |  |
| Devon Jones | Mala Murthy Lab, Sebastian Seung Lab | 1,296 |  |
| Annalena Oswald | Marion Silies Lab | 1,214 |  |
| Lucas Encarnacion-Rivera | Mala Murthy Lab | 1,164 |  |
| Akanksha Jadia | Greg Jefferis Lab | 1,141 |  |
| Nik Drummond | Alexander Borst Lab | 1,104 |  |
| Leonie Walter | Mathias Wernet Lab | 1,102 |  |
| Xin Zhong | Mathias Wernet Lab | 1,083 |  |
| Benjamin Gorko | Sung Soo Kim Lab | 1,064 |  |
| Jonas Chojetzki | Marion Silies Lab | 1,060 |  |
| Fernando J Figueroa Santiago | Seeds Hampel Lab | 1,049 |  |
| István Taisz | Greg Jefferis Lab | 1,043 |  |
| Urja Verma | Greg Jefferis Lab | 1,033 |  |
| Annika Bast | Marion Silies Lab | 1,031 |  |
| Ibrahim Tastekin | Carlos Ribeiro Lab | 1,025 | Tracing taste peg gustatory neurons and downstream neurons |
| Sandeep Kumar | Mala Murthy Lab | 987 |  |
| Yuta Mabuchi | Nilay Yapici Lab | 963 |  |
| Nick Byrne | Wei-Chung Lee Lab | 951 |  |
| Edda Kunze | Gerit Linneweber Lab | 907 |  |
| Thomas Crahan | Sung Soo Kim Lab | 901 |  |
| Hewhoamareismyself (Ryan Margossian) | Flyers (citizen scientist) | 874 |  |
| Maria Ioannidou | Marion Silies Lab | 848 |  |
| Iliyan Georgiev | Flyers (citizen scientist) | 825 |  |
| Fabianna Szorenyi | Seeds Hampel Lab | 817 |  |
| Atsuko Adachi | Richard Mann Lab, Rudy Behnia Lab | 745 |  |
| Tomke Stuerner | Greg Jefferis Lab | 736 |  |
| Minsik Yun | Young-Joon Kim Lab | 625 |  |
| Andrearwen | Flyers (citizen scientist) | 607 |  |
| Robert Turnbull | Greg Jefferis Lab | 586 |  |
| Eleni Samara | Alexander Borst Lab | 546 |  |
| Sebastian Molina-Obando | Marion Silies Lab | 545 |  |
| Alexander Thomson | Janelia, Michael Reiser Lab | 527 |  |
| a5hm0r | Flyers (citizen scientist) | 516 |  |
| Lena Lörsch | Marion Silies Lab | 497 |  |
| Connor Laughland | Janelia, Michael Reiser Lab | 469 |  |
| Suchetana B. Dutta | Bassem Hassan Lab | 458 |  |
| Paula Guiomar Alarcón de Antón | Mathias Wernet Lab | 426 |  |
| Patricia Pujols | Seeds Hampel Lab | 423 |  |
| Binglin Huang | Sung Soo Kim Lab | 423 |  |
| Kenneth J. Colodner | Ken Colodner Lab | 421 |  |
| Isabel Haber | Rachel Wilson Lab | 392 |  |
| Albert Lin | Mala Murthy Lab | 362 |  |
| Daniel T. Choe | Jinseop Kim Lab | 340 |  |
| Alexander Shakeel Bates | Greg Jefferis Lab, Rachel Wilson Lab | 340 |  |
| Veronika Lukyanova | Jenny Read Lab | 337 |  |
| Marta Costa | Greg Jefferis Lab | 334 |  |
| Zequan Liu | Xueying "Snow" Wang | 317 |  |
| Haley Croke | Katie von Reyn Lab | 308 |  |
| Gizem Sancer | Mathias Wernet Lab | 308 |  |
| Tatsuo Okubo | Rachel Wilson Lab | 306 |  |
| Miriam A. Flynn | Janelia, Michael Reiser Lab | 297 |  |
| Meghan Laturney | Kristin Scott Lab | 274 |  |
| Benjamin Bargeron | Salil Bidaye Lab | 273 |  |
| Davi D. Bock | Davi Bock Lab | 255 |  |
| Hyunsoo Yim | Jinseop Kim Lab | 240 |  |
| Anh Duc Le | Denise Garcia Lab | 237 |  |
| Seungyun Yu | Jae Young Kwon Lab | 224 |  |
| Yeonju Nam | Jinseop Kim Lab | 221 |  |
| Mavil | Flyers (citizen scientist) | 217 |  |
| Audrey Francis | Gaby Maimon Lab | 196 |  |
| Jesse Gayk | Greg Jefferis Lab | 195 |  |
| Zepeng Yao | Kristin Scott Lab | 194 |  |
| Sommer S. Huntress | Ken Colodner Lab | 192 |  |
| Carolina Manyari-Diaz | Salil Bidaye Lab | 191 |  |
| Raquel Barajas | Carlos Ribeiro Lab | 186 |  |
| Mindy Kim | Wei-Chung Lee Lab | 185 |  |
| Burak Gür | Marion Silies Lab | 182 |  |
| Nils Reinhard | Charlotte Helfrich-Forster Lab | 177 | Tracing of clock and AME neurons |
| Amanda Abusaif | Kristin Scott Lab | 176 |  |
| Anna Li | Rachel Wilson Lab | 173 |  |
| Sven Dorkenwald | Sebastian Seung Lab | 171 |  |
| Fred W Wolf | Fred Wolf Lab | 163 |  |
| Keehyun Park | Jae Young Kwon Lab | 155 |  |
| Xinyue Cui | Nilay Yapici Lab | 152 |  |
| Haein Kim | Nilay Yapici Lab | 145 |  |
| Georgia Dempsey | Greg Jefferis Lab, Scott Waddell Lab | 143 |  |
| Alan Mathew | Greg Jefferis Lab | 141 |  |
| Jinseong Kim | Jinseop Kim Lab | 141 |  |
| Taewan Kim | Jinseop Kim Lab | 135 |  |
| Guan-ting Wu | National Hualien Senior High School | 124 |  |
| Margarida Brotas | Eugenia Chiappe Lab | 112 |  |
| Cheng-hao Zhang | National Hualien Senior High School | 109 |  |
| Philip K. Shiu | Kristin Scott Lab | 108 |  |
| Shanice Bailey | Greg Jefferis Lab | 102 |  |

**Supplementary Table 1 | Number of proofreading edits by consortium members.** Only members with ≥100 edits are shown.

| **Name** | **Lab Affiliation** | **total labels** | **specific contributions** |
| --- | --- | --- | --- |
| Krzysztof Kruk | Flyers (citizen scientist) | 44,579 |  |
| Volker Hartenstein | Volker Hartenstein Lab | 13,762 |  |
| Alexander Shakeel Bates | Greg Jefferis Lab, Rachel Wilson Lab | 11,260 |  |
| Sven Dorkenwald | Sebastian Seung Lab | 6,375 |  |
| Katharina Eichler | Greg Jefferis Lab, Seeds Hampel Lab | 6,366 |  |
| Ben Silverman | Mala Murthy Lab, Sebastian Seung Lab | 4,624 |  |
| Doug Bland | Mala Murthy Lab, Sebastian Seung Lab | 4,182 |  |
| Philipp Schlegel | Greg Jefferis Lab | 3,754 |  |
| Austin T Burke | Mala Murthy Lab, Sebastian Seung Lab | 3,242 |  |
| Jay Gager | Mala Murthy Lab, Sebastian Seung Lab | 3,175 |  |
| annkri (Anne Kristiansen) | Flyers (citizen scientist) | 3,057 |  |
| Stefanie Hampel | Seeds Hampel Lab | 2,859 |  |
| AzureJay (Jaime Skelton) | Flyers (citizen scientist) | 2,845 |  |
| Celia David | Mala Murthy Lab, Sebastian Seung Lab | 2,685 |  |
| Kyle Patrick Willie | Mala Murthy Lab, Sebastian Seung Lab | 2,566 |  |
| David Deutsch | Mala Murthy Lab | 2,549 |  |
| Kaiyu Wang | Barry Dickson Lab | 2,443 |  |
| Yijie Yin | Greg Jefferis Lab | 2,354 |  |
| Thomas Stocks | Flyers (citizen scientist) | 1,964 |  |
| Dustin Garner | Sung Soo Kim Lab | 1,788 |  |
| James Hebditch | Mala Murthy Lab, Sebastian Seung Lab | 1,667 |  |
| Wolf Huetteroth | Wolf Huetteroth Lab | 1,524 |  |
| Nashra Hadjerol | Mala Murthy Lab, Sebastian Seung Lab | 1,315 |  |
| Ryan Willie | Mala Murthy Lab, Sebastian Seung Lab | 1,308 |  |
| Joshua Bañez | Mala Murthy Lab, Sebastian Seung Lab | 1,160 |  |
| Rey Adrian Candilada | Mala Murthy Lab, Sebastian Seung Lab | 1,117 |  |
| Amalia Braun | Alexander Borst Lab | 1,107 |  |
| Kendrick Joules Vinson | Mala Murthy Lab, Sebastian Seung Lab | 1,058 |  |
| Remer Tancontian | Mala Murthy Lab, Sebastian Seung Lab | 1,029 |  |
| Lena Lörsch | Marion Silies Lab | 1,009 |  |
| Gizem Sancer | Mathias Wernet Lab | 942 |  |
| Regine Salem | Mala Murthy Lab, Sebastian Seung Lab | 914 |  |
| Jenna Joroff | Wei-Chung Lee Lab | 900 |  |
| Zairene Lenizo | Mala Murthy Lab, Sebastian Seung Lab | 738 |  |
| Gregory S.X.E. Jefferis | Greg Jefferis Lab | 716 |  |
| Sebastian Molina-Obando | Marion Silies Lab | 676 |  |
| Christa Baker | Mala Murthy Lab | 622 |  |
| Jet Ivan Dolorosa | Mala Murthy Lab, Sebastian Seung Lab | 621 |  |
| Claire E. McKellar | Mala Murthy Lab, Sebastian Seung Lab | 612 |  |
| John Anthony Ocho | Mala Murthy Lab, Sebastian Seung Lab | 570 |  |
| Markus William Pleijzier | Greg Jefferis Lab | 541 | Reconstruction of Mushroom Body neurons, Lateral Horn Neurons and Lateral Horn Centrifugal Neurons |
| Christopher Dunne | Greg Jefferis Lab | 517 |  |
| Szi-chieh Yu | Mala Murthy Lab, Sebastian Seung Lab | 509 |  |
| Ariel Dagohoy | Mala Murthy Lab, Sebastian Seung Lab | 493 |  |
| Márcia dos Santos | Greg Jefferis Lab | 448 |  |
| Varun Sane | Greg Jefferis Lab | 442 |  |
| Quinn Vanderbeck | Rachel Wilson Lab | 424 |  |
| Lucia Kmecova | Seeds Hampel Lab | 412 |  |
| Steven Calle | Seeds Hampel Lab | 408 |  |
| Marion Silies | Marion Silies | 379 |  |
| Philip K. Shiu | Kristin Scott Lab | 321 |  |
| Eva Munnelly | Greg Jefferis Lab | 312 |  |
| Marina Gkantia | Greg Jefferis Lab | 300 |  |
| Jonas Chojetzki | Marion Silies Lab | 254 |  |
| Jonas Chojetzki | Marion Silies | 254 |  |
| Farzaan Salman | Andrew Dacks Lab | 234 |  |
| Matt Collie | Rachel Wilson Lab | 223 |  |
| Annika Bast | Marion Silies Lab | 193 |  |
| Marissa Sorek | Mala Murthy Lab, Sebastian Seung Lab | 179 |  |
| Kenneth J. Colodner | Ken Colodner Lab | 161 |  |
| Gerit A. Linneweber | Gerit Linneweber Lab | 157 |  |
| Mareike Selcho | Mareike Selcho Lab | 155 |  |
| TR77 | Flyers (citizen scientist) | 147 |  |
| Maria Ioannidou | Marion Silies Lab | 144 |  |
| Nils Reinhard | Charlotte Helfrich-Forster Lab | 134 | Identification of clock and AME neurons |
| Megan Wang | Mala Murthy Lab | 130 |  |
| Meet Zandawala | Zandawala Lab | 128 | Identification of peptidergic and gustatory neurons |
| Lucy Houghton | Sung Soo Kim Lab | 123 |  |
| Mert Erginkaya | Eugenia Chiappe Lab | 110 | Identification and annotation of several cell types in the IPS-SPS-GNG regions |
| Benjamin Gorko | Sung Soo Kim Lab | 107 |  |
| Nseraf | Flyers (citizen scientist) | 107 |  |
| Haein Kim | Nilay Yapici Lab | 72 |  |
| Minsik Yun | Young-Joon Kim Lab | 71 |  |
| Damian Demarest | Michael Pankratz Lab | 70 |  |
| István Taisz | Greg Jefferis Lab | 66 |  |
| Andrea Sandoval | Kristin Scott Lab | 58 |  |
| Anthony Moreno-Sanchez | Katie von Reyn Lab | 56 |  |
| Diego A. Pacheco | Mala Murthy Lab | 55 |  |
| Zhihao Zheng | Sebastian Seung Lab | 51 |  |
| Burak Gür | Marion Silies Lab | 49 |  |
| Benjamin Bargeron | Salil Bidaye Lab | 47 |  |
| Sandeep Kumar | Mala Murthy Lab | 40 |  |
| Tansy Yang | Janelia | 37 |  |
| Amanda González-Segarra | Kristin Scott Lab | 36 |  |
| Nino Mancini | Salil Bidaye Lab | 33 |  |
| Gianna Vitelli | Salil Bidaye Lab | 29 |  |
| Feng Li | Janelia | 26 |  |
| Joanna Eckhardt | Mala Murthy Lab | 26 |  |
| Shuo Cao | David Anderson Lab | 24 |  |
| Álvaro Sanz Díez | Rudy Behnia Lab | 24 |  |
| Haley Croke | Katie von Reyn Lab | 22 |  |
| Stéphane Noselli | Stéphane Noselli Lab | 20 |  |
| Nelsie Panes | Mala Murthy Lab, Sebastian Seung Lab | 18 |  |
| Gabriella R. Sterne | Gabriella Sterne Lab | 18 |  |
| Kate Maier | Salil Bidaye Lab | 16 |  |
| Amy R Sterling | Sebastian Seung Lab | 15 |  |
| Allien Mae Gogo | Mala Murthy Lab, Sebastian Seung Lab | 13 |  |
| Yuta Mabuchi | Nilay Yapici Lab | 12 |  |
| Cathy Pilapil | Mala Murthy Lab, Sebastian Seung Lab | 11 |  |
| Zepeng Yao | Kristin Scott Lab | 10 |  |
| Alexander Del Toro | Kristin Scott Lab | 10 |  |
| Jacquilyn Laude | Mala Murthy Lab, Sebastian Seung Lab | 10 |  |
| Lucas Encarnacion-Rivera | Mala Murthy Lab | 10 |  |

**Supplementary Table 2 | Number of annotations by consortium members.** Only members with ≥10 annotations are shown.

| **First author** | **Title** | **Year** | **DOI** |
| --- | --- | --- | --- |
| Deutsch et al. | The neural basis for a persistent internal state in Drosophila females | 2020 | <https://doi.org/10.7554/eLife.59502> |
| Schlegel et al. | Information flow, cell types and stereotypy in a full olfactory connectome | 2021 | <https://doi.org/10.7554/eLife.66018> |
| Sterne et al. | Classification and genetic targeting of cell types in the primary taste and premotor center of the adult Drosophila brain | 2021 | <https://doi.org/10.7554/eLife.71679> |
| Kind et al. | Synaptic targets of photoreceptors specialized to detect color and skylight polarization in Drosophila | 2021 | <https://doi.org/10.7554/eLife.71858> |
| Zhao et al. | Eye structure shapes neuron function in Drosophila motion vision | 2022 | <https://doi.org/10.1101/2022.12.14.520178> |
| Zheng et al. | Structured sampling of olfactory input by the fly mushroom body | 2022 | <https://doi.org/10.1016/j.cub.2022.06.031> |
| Shiu et al. | Taste quality and hunger interactions in a feeding sensorimotor circuit | 2022 | <https://doi.org/10.7554/eLife.79887.sa0> |
| Chou et al. | Mating-driven variability in olfactory local interneuron wiring | 2022 | <https://doi.org/10.1126/sciadv.abm7723> |
| Israel et al. | Olfactory stimuli and moonwalker SEZ neurons can drive backward locomotion in Drosophila | 2022 | <https://doi.org/10.1016/j.cub.2022.01.035> |
| Task et al. | Chemoreceptor co-expression in Drosophila melanogaster olfactory neurons | 2022 | <https://doi.org/10.7554/eLife.72599> |
| Schlegel et al. | Whole-brain annotation and multi-connectome cell typing quantifies circuit stereotypy in Drosophila | 2023 | <https://doi.org/10.1101/2023.06.27.546055> |
| Matsliah et al. | Neuronal "parts list" and wiring diagram for a visual system | 2023 | <https://doi.org/10.1101/2023.10.12.562119> |
| Jacobs et al. | Overlap and divergence of neural circuits mediating distinct behavioral responses to sugar | 2023 | <https://doi.org/10.1101/2023.10.01.560401> |
| Reinhard et al. | Synaptic and peptidergic connectomes of the Drosophila circadian clock | 2023 | <https://doi.org/10.1101/2023.09.11.557222> |
| Frantzmann et al. | Neuronal correlates of time integration into memories | 2023 | <https://doi.org/10.1101/2023.09.12.557375> |
| Braun et al. | Networks of descending neurons transform command-like signals into population-based behavioral control | 2023 | <https://doi.org/10.1101/2023.09.11.557103> |
| Ros et al. | Descending control and regulation of spontaneous flight turns in Drosophila | 2023 | <https://doi.org/10.1016/j.cub.2023.12.047> |
| Shiu et al. | A leaky integrate-and-fire computational model based on the connectome of the entire adult Drosophila brain reveals insights into sensorimotor processing | 2023 | <https://doi.org/10.1101/2023.09.06.555791> |
| González-Segarra et. al. | Hunger- and thirst-sensing neurons modulate a neuroendocrine network to coordinate sugar and water ingestion | 2023 | <https://doi.org/10.7554/eLife.88143.3> |
| Braun et al. | Disynaptic inhibition shapes tuning of OFF-motion detectors in Drosophila | 2023 | <https://doi.org/10.1016/j.cub.2023.05.007> |
| Christenson et al. | Hue selectivity from recurrent circuitry in Drosophila | 2023 | <https://doi.org/10.1101/2023.07.12.548573> |
| Eckstein et al. | Neurotransmitter Classification from Electron Microscopy Images at Synaptic Sites in Drosophila Melanogaster | 2023 | <https://doi.org/10.1101/2020.06.12.148775> |
| Mabuchi et al. | Visual Feedback Neurons Fine-Tune Drosophila Male Courtship via GABA-Mediated Inhibition | 2023 | <https://doi.org/10.1016/j.cub.2023.08.034> |
| Eichler et al. | Somatotopic organization among parallel sensory pathways that promote a grooming sequence in Drosophila | 2023 | <https://doi.org/10.7554/eLife.87602.1> |
| Baker et al. | Neural network organization for courtship-song feature detection in Drosophila | 2023 | <https://doi.org/10.1016/j.cub.2022.06.019> |
| Sapkal et al. | Neural circuit mechanisms underlying context-specific halting in Drosophila | 2023 | <https://doi.org/10.1101/2023.09.25.559438> |
| Cornean et al. | Heterogeneity of synaptic connectivity in the fly visual system | 2023 | <https://doi.org/10.1101/2023.08.29.555204> |
| Ganguly et al. | Diversity of visual inputs to Kenyon cells of the Drosophila mushroom body | 2023 | <https://doi.org/10.1101/2023.10.12.561793> |
| Zhao et al. | A comprehensive neuroanatomical survey of the Drosophila Lobula Plate Tangential Neurons with predictions for their optic flow sensitivity | 2023 | <https://doi.org/10.1101/2023.10.16.562634> |
| Yang et al. | Fine-grained descending control of steering in walking Drosophila. | 2023 | <https://doi.org/10.1101/2023.10.15.562426> |
| Pospisil et al. | From connectome to effectome: learning the causal interaction map of the fly brain | 2023 | <https://doi.org/10.1101/2023.10.31.564922> |
| Seung | Insights into vision from interpretation of a neuronal wiring diagram | 2023 | <https://doi.org/10.1101/2023.11.15.567126> |
| Garner et al. | Connectomic reconstruction predicts the functional organization of visual inputs to the navigation center of the Drosophila brain | 2023 | <https://doi.org/10.1101/2023.11.29.569241> |
| Lin et al. | Network Statistics of the Whole-Brain Connectome of Drosophila | 2023 | <https://doi.org/10.1101/2023.07.29.551086> |

**Supplementary Table 3 | Publications utilizing FlyWire before publication**

**Supplementary Video 1 | Rendering of all neurons in the fly brain.** All neurons in the fly brain are shown in different colors. Then, neurons belonging to each super class (Fig. 2 a-c) are shown.

**Supplementary Video 2 | Neuropils of the fly brain.** All neuropils are shown. See Ext. Data Fig. 1 for color key.
