# Supplementary material for: Neuronal wiring diagram of an adult brain
Source: Nature. 2024 Oct 2;634(8032):124–38. doi: 10.1038/s41586-024-07558-y (PMC11446842; doi:10.1038/s41586-024-07558-y)
Supplement: Supplementary file 3 — Full list of authors and affiliations of the FlyWire Consortium. [file 41586_2024_7558_MOESM3_ESM.pdf]

Supplementary Note

FlyWire Consortium

Krzysztof Kruk[3], Doug Bland[1], Zairene Lenizo[16], Austin T. Burke[1], Kyle Patrick Willie[1], Alexander Shakeel Bates[4,5,12,13], Nikitas Serafetinidis[3], Nashra Hadjerol[16], Ryan Willie[1], Katharina Eichler[5], Ben Silverman[1], John Anthony Ocho[16], Joshua Bañez[16], Yijie Yin[5], Rey Adrian Candilada[16], Sven Dorkenwald[1,2], Jay Gager[1], Anne Kristiansen[3], Nelsie Panes[16], Arti Yadav[17], Remer Tancontian[16], Shirleyjoy Serona[16], Jet Ivan Dolorosa[16], Kendrick Joules Vinson[16], Dustin Garner[18], Regine Salem[16], Ariel Dagohoy[16], Philipp Schlegel[4,5], Jaime Skelton[3], Mendell Lopez[16], Laia Serratosa Capdevila[5], Griffin Badalamente[5], Thomas Stocks[3], Anjali Pandey[17], Darrel Jay Akiatan[16], James Hebditch[1], Celia David[1], Dharini Sapkal[17], Shaina Mae Monungolh[16], Varun Sane[5], Mark Lloyd Pielago[16], Miguel Albero[16], Jacquilyn Laude[16], Márcia dos Santos[5], David Deutsch[1,9], Zeba Vohra[17], Kaiyu Wang[14], Allien Mae Gogo[16], Emil Kind[19], Alvin Josh Mandahay[16], Chereb Martinez[16], John David Asis[16], Chitra Nair[17], Dhwani Patel[17], Marchan Manaytay[16], Imaan F. M. Tamimi[5], Clyde Angelo Lim[16], Philip Lenard Ampo[16], Michelle Darapan Pantujan[16], Alexandre Javier[5], Daril Bautista[16], Rashmita Rana[17], Jansen Seguido[16], Bhargavi Parmar[17], John Clyde Saguimpa[16], Merlin Moore[1], Markus William Pleijzier[4], Mark Larson[20], Joseph Hsu[5], Itisha Joshi[17], Dhara Kakadiya[17], Amalia Braun[21], Cathy Pilapil[16], Marina Gkantia[5], Kaushik Parmar[17], Quinn Vanderbeck[12], Claire E. McKellar[1], Irene Salgarella[5], Christopher Dunne[5], Eva Munnelly[5], Chan Hyuk Kang[22], Lena Lörsch[27], Jinmook Lee[22], Lucia Kmecova[24], Gizem Sancer[23], Christa Baker[1], Szi-chieh Yu[1], Jenna Joroff[12], Steven Calle[24], Yashvi Patel[17], Olivia Sato[20], Siqi Fang[5], Janice Salocot[16], Farzaan Salman[26], Sebastian Molina-Obando[27], Paul Brooks[5], Mai Bui[25], Matthew Lichtenberger[3], Edmark Tamboboy[16], Katie Molloy[20], Alexis E Santana-Cruz[24], Anthony Hernandez[3], Seongbong Yu[22], Marissa Sorek[1,3], Arzoo Diwan[17], Monika Patel[17], Travis R. Aiken[3], Sarah Morejohn[1], Sanna Koskela[14], Tansy Yang[14], Daniel Lehmann[3], Jonas Chojetzki[27], Sangeeta Sisodiya[17], Selden Koolman[1], Philip K. Shiu[28], Sky Cho[25], Annika Bast[27], Brian Reicher[20], Marlon Blanquart[5], Lucy Houghton[18], Hyungjun Choi[22], Maria Ioannidou[27], Matt Collie[20], Joanna Eckhardt[1], Benjamin Gorko[18], Li Guo[18], Zhihao Zheng[1], Alisa Poh[29], Marina Lin[25], István Taisz[4], Wes Murfin[53], Álvaro Sanz Díez[37], Nils Reinhard[30], Peter Gibb[12], Nidhi Patel[17], Sandeep Kumar[1], Minsik Yun[31], Megan Wang[1], Devon Jones[1], Lucas Encarnacion-Rivera[32], Annalena Oswald[27], Akanksha Jadia[17], Mert Erginkaya[33], Nik Drummond[5], Leonie Walter[19], Ibrahim Tastekin[33], Xin Zhong[19], Yuta Mabuchi[34], Fernando J. Figueroa Santiago[24], Urja Verma[17], Nick Byrne[20], Edda Kunze[19], Thomas Crahan[18], Ryan Margossian[3], Haein Kim[34], Iliyan Georgiev[3], Fabianna Szorenyi[24], Atsuko Adachi[37], Benjamin Bargeron[35], Tomke Stuerner[4,5], Damian Demarest[36], Burak Gür[27], Andrea N. Becker[3], Robert Turnbull[5],

Ashley Morren[3], Andrea Sandoval[28], Anthony Moreno-Sanchez[38], Diego A. Pacheco[12], Eleni Samara[21], Haley Croke[38], Alexander Thomson[14], Connor Laughland[14], Suchetana B. Dutta[19], Paula Guiomar Alarcón de Antón[19], Binglin Huang[18], Patricia Pujols[24], Isabel Haber[20], Amanda González-Segarra[28], Albert Lin[1,6], Daniel T. Choe[39], Veronika Lukyanova[40], Marta Costa[5], Nino Mancini[35], Zequan Liu[41], Tatsuo Okubo[12], Miriam A. Flynn[14], Gianna Vitelli[35], Meghan Laturney[28], Feng Li[14], Shuo Cao[42], Carolina Manyari-Diaz[35], Hyunsoo Yim[39], Anh Duc Le[38], Kate Maier[35], Seungyun Yu[22], Yeonju Nam[22], Daniel Bąba[3], Amanda Abusaif[28], Audrey Francis[43], Jesse Gayk[17], Sommer S. Huntress[44], Raquel Barajas[33], Mindy Kim[20], Xinyue Cui[34], Amy R. Sterling[1,3], Gabriella R. Sterne[28], Anna Li[12], Keehyun Park[22], Georgia Dempsey[5], Alan Mathew[5], Jinseong Kim[22], Taewan Kim[22], Guan-ting Wu[45], Serene Dhawan[46], Margarida Brotas[33], Cheng-hao Zhang[45], Shanice Bailey[5], Alexander Del Toro[28], Arie Matsliah[1], Kisuk Lee[1,10], Thomas Macrina[1,2], Casey Schneider-Mizell[7], Sergiy Popovych[1,2], Oluwaseun Ogedengbe[1], Runzhe Yang[1], Akhilesh Halageri[1], Will Silversmith[1], Stephan Gerhard[47], Andrew Champion[4,5], Nils Eckstein[14], Dodam Ih[1], Nico Kemnitz[1], Manuel Castro[1], Zhen Jia[1], Jingpeng Wu[1], Eric Mitchell[1], Barak Nehoran[1,2], Shang Mu[1], J. Alexander Bae[1,11], Ran Lu[1], Eric Perlman[8], Ryan Morey[1], Kai Kuehner[1], Derrick Brittain[7], Chris S. Jordan[1], David J. Anderson[42], Rudy Behnia[37], Salil S. Bidaye[35], Davi D. Bock[15], Alexander Borst[21], Eugenia Chiappe[33], Forrest Collman[7], Kenneth J. Colodner[44], Andrew Dacks[26], Barry Dickson[14], Jan Funke[14], Denise Garcia[38], Stefanie Hampel[24], Volker Hartenstein[48], Bassem Hassan[19], Charlotte Helfrich-Forster[30], Wolf Huetteroth[49], Gregory S.X.E. Jefferis[4,5], Jinseop Kim[22], Sung Soo Kim[18], Young-Joon Kim[31], Jae Young Kwon[22], Wei-Chung Lee[12], Gerit A. Linneweber[19], Gaby Maimon[43], Richard Mann[37], Mala Murthy[1], Stéphane Noselli[52], Michael Pankratz[36], Lucia Prieto-Godino[46], Jenny Read[40], Michael Reiser[14], Katie von Reyn[38], Carlos Ribeiro[33], Kristin Scott[28], Andrew M. Seeds[24], Mareike Selcho[49], H. Sebastian Seung[1,2], Marion Silies[27], Julie Simpson[18], Scott Waddell[54], Mathias F. Wernet[19], Rachel I. Wilson[12], Fred W. Wolf[50], Zepeng Yao[51], Nilay Yapici[34], Meet Zandawala[30]

[1]Princeton Neuroscience Institute, Princeton University, Princeton, USA
[2]Computer Science Department, Princeton University, Princeton, USA
[3]Eyewire, Boston, MA, USA
[4]Neurobiology Division, MRC Laboratory of Molecular Biology, Cambridge, UK
[5]Drosophila Connectomics Group, Department of Zoology, University of Cambridge, Cambridge, UK
[6]Center for the Physics of Biological Function, Princeton University, Princeton, USA
[7]Allen Institute for Brain Science, Seattle, USA
[8]Yikes LLC, Baltimore, USA
[9]Department of Neurobiology, University of Haifa, Haifa, Israel
[10]Brain & Cognitive Sciences Department, Massachusetts Institute of Technology, Cambridge, USA
[11]Electrical and Computer Engineering Department, Princeton University, Princeton, USA
[12]Harvard Medical School, Boston, USA
[13]Centre for Neural Circuits and Behaviour, The University of Oxford, Oxford, UK
[14]Janelia Research Campus, Howard Hughes Medical Institute, Ashburn, USA
[15]Department of Neurological Sciences, Larner College of Medicine, University of Vermont, Burlington, USA
[16]SixEleven, Davao City, Philippines
[17]ariadne.ai ag, Buchrain, Switzerland
[18]University of California, Santa Barbara, USA
[19]Freie Universität Berlin, Berlin, Germany

[20]Harvard, Boston, USA
[21]Department Circuits-Computation-Models, Max Planck Institute for Biological Intelligence, Planegg, Germany
[22]Sungkyunkwan University, Seoul, South Korea
[23]Department of Neuroscience, Yale University, New Haven, USA
[24]Institute of Neurobiology, University of Puerto Rico Medical Sciences Campus, San Juan, Puerto Rico
[25]Program in Neuroscience and Behavior, Mount Holyoke College, South Hadley, USA
[26]Department of Biology, West Virginia University, Morgantown, USA
[27]Johannes-Gutenberg University Mainz, Mainz, Germany
[28]University of California, Berkeley, USA
[29]University of Queensland, Brisbane, Australia
[30]Julius-Maximilians-Universität Würzburg, Würzburg, Germany
[31]Gwangju Institute of Science and Technology, Gwangju, South Korea
[32]Stanford University School of Medicine, Stanford, USA
[33]Champalimaud Foundation, Lisbon, Portugal
[34]Cornell University, Ithaca, USA
[35]Max Planck Florida Institute for Neuroscience, Jupiter, USA
[36]University of Bonn, Bonn, Germany
[37]Zuckerman Institute, Columbia University, New York, USA
[38]Drexel, Philadelphia, USA
[39]Seoul National University, Seoul, South Korea
[40]Newcastle University, Newcastle, UK
[41]RWTH Aachen University, Aachen, Germany
[42]Caltech, Pasadena, USA
[43]Rockefeller University, New York, USA
[44]Mount Holyoke College, South Hadley, USA
[45]National Hualien Senior High School, Hualien, Taiwan
[46]The Francis Crick Institute, London, UK
[47]Aware LLC, Zurich, Switzerland
[48]University of California, Los Angeles, USA
[49]Institute of Biology, Leipzig University, Leipzig, Germany
[50]University of California, Merced, USA
[51]University of Florida, Gainesville, USA
[52]Université Côte d’Azur, CNRS, Inserm, iBV, Nice, France
[53]Retired MD-PhD, Fort Collins, USA
[54]University of Oxford, Oxford, UK
