# Supplementary material for: Neuronal wiring diagram of an adult brain
Source: Nature. 2024 Oct 2;634(8032):124–38. doi: 10.1038/s41586-024-07558-y (PMC11446842; doi:10.1038/s41586-024-07558-y)
Supplement: Supplementary file 4 — Full list of Eyewire authors and their affiliations. [file 41586_2024_7558_MOESM4_ESM.pdf]

Supplementary Note

Eyewire authors

Krzysztof Kruk[3], Nikitas Serafetinidis (Nseraf)[3], Anne Kristiansen[3], Jaime Skelton (AzureJay)[3], Thomas Stocks[3], Matthew Lichtenberger (JousterL)[3], Anthony Hernandez (Kfay)[3], Marissa Sorek[1,3], Travis R. Aiken (TR77)[3], Daniel Lehmann (bl4ckscor3)[3], Ryan Margossian (Hewhoamareismyself)[3], Iliyan Georgiev[3], Andrea N. Becker[3], Ashley Morren (a5hm0r)[3], Daniel Bąba (Mavil)[3], Amy R. Sterling[1,3]

[1]Princeton Neuroscience Institute, Princeton University, Princeton, NJ, USA
[3]Eyewire, Boston, MA, USA
